# Ecological and Pharmacological Activities of Polybrominated Diphenyl Ethers (PBDEs) from the Indonesian Marine Sponge *Lamellodysidea herbacea*

**DOI:** 10.3390/md19110611

**Published:** 2021-10-27

**Authors:** Muhammad R. Faisal, Matthias Y. Kellermann, Sven Rohde, Masteria Y. Putra, Tutik Murniasih, Chandra Risdian, Kathrin I. Mohr, Joachim Wink, Dimas F. Praditya, Eike Steinmann, Matthias Köck, Peter J. Schupp

**Affiliations:** 1 Environmental Biochemistry, Institute of Chemistry and Biology of the Marine Environment (ICBM), Carl-von-Ossietzky University of Oldenburg, Schleusenstr. 1, 26382 Wilhelmshaven, Germany; muhammad.reza.faisal@uni-oldenburg.de (M.R.F.); matthias.kellerman@uni-oldenburg.de (M.Y.K.); sven.rohde@uni-oldenburg.de (S.R.); 2 Research Center for Biotechnology, Research Organization for Life Science, National Research and Innovation Agency (BRIN), Cibinong 16911, Indonesia; mast001@lipi.go.id (M.Y.P.); tuti003@brin.go.id (T.M.); dima007@lipi.go.id (D.F.P.); 3 Microbial Strain Collection (MISG), Helmholtz Centre for Infection Research, Inhoffenstraße 7, 38124 Braunschweig, Germany; chandra.risdian@helmholtz-hzi.de (C.R.); kathrinmohr4@gmail.com (K.I.M.); joachim.wink@helmholtz-hzi.de (J.W.); 4 Research Unit for Clean Technology, Indonesian Institute of Sciences (LIPI), Bandung 40135, Indonesia; 5 TWINCORE-Centre for Experimental and Clinical Infection Research, Institute of Experimental Virology, Feodor-Lynen-Str. 7–9, 30625 Hannover, Germany; eike.steinmann@ruhr-uni-bochum.de; 6 Department of Molecular and Medical Virology, Ruhr-University Bochum, 44801 Bochum, Germany; 7 Alfred Wegener Institute, Helmholtz Centre for Polar and Marine Research, Am Handelshafen 12, 27570 Bremerhaven, Germany; matthias.koeck@awi.de; 8 Helmholtz Institute for Functional Marine Biodiversity (HIFMB), University of Oldenburg, Ammerländer Heerstraße 231, 26129 Oldenburg, Germany

**Keywords:** demospongiae, chemical defense, palatability, antifungal, inhibitory, PBDEs

## Abstract

Two known Polybrominated Diphenyl Ethers (PBDEs), 3,4,5-tribromo-2-(2′,4′-dibromophenoxy)phenol (**1d**) and 3,4,5,6-tetrabromo-2-(2′,4′-dibromophenoxy)phenol (**2b**), were isolated from the Indonesian marine sponge *Lamellodysidea herbacea*. The structure was confirmed using ^13^C chemical shift average deviation and was compared to the predicted structures and recorded chemical shifts in previous studies. We found a wide range of bioactivities from the organic crude extract, such as (1) a strong deterrence against the generalist pufferfish *Canthigaster solandri*, (2) potent inhibition against environmental and human pathogenic bacterial and fungal strains, and (3) the inhibition of the Hepatitis C Virus (HCV). The addition of a bromine atom into the A-ring of compound **2b** resulted in higher fish feeding deterrence compared to compound **1d**. On the contrary, compound **2b** showed only more potent inhibition against the Gram-negative bacteria *Rhodotorula glutinis* (MIC 2.1 μg/mL), while compound **1d** showed more powerful inhibition against the other human pathogenic bacteria and fungi. The first report of a chemical defense by compounds **1d** and **2b** against fish feeding and environmental relevant bacteria, especially pathogenic bacteria, might be one reason for the widespread occurrence of the shallow water sponge *Lamellodysidea herbacea* in Indonesia and the Indo-Pacific.

## 1. Introduction

Sponges are known to harbour a huge variety of secondary metabolites, which could potentially be important leads in drug development [1]. Reported compound classes include but are not limited to peptides [2,3,4], pyridines [5], diterpenoids [6], alkaloids [7], lectins [8], carotenoids [9], steroids [10,11], and sterols [12] with reported bioactivities ranging from pharmaceutical (antimicrobial, antitumor, anti-inflammatory ATPase activator, cytotoxicity, neuroprotective) to ecological (feeding deterrent, antifouling). The continued discovery of novel bioactive marine natural products (MNPs) has been well documented in detailed review articles from *Natural Product Reports* (i.e., [13,14,15]).

*Lamellodysidea herbacea* is a common sponge of shallow coral reef habitats, especially reef flats, back-reef, and lagoon-type habitats, throughout the Indo-Pacific. There have likely been several synonyms in the literature for this species, as it has undergone several taxonomic revisions. This species was first described as *Spongelia herbacea* (Keller, 1889), which was later revised to *Dysidea herbacea* (Keller, 1889), and is now accepted as *Lamellodysidea herbacea* (Keller, 1889) [16]. Having several taxonomic revisions also makes it harder to keep track of reported compounds or compound classes for particular species or even higher taxonomic ranks such as genus or family [17]. Based on previous publications, including the synonym genera, *Lamellodysidea* should be regarded as a sponge genus containing a wide variety of secondary metabolites divided into three distinct classes, namely terpenoids, polychlorinated amino-acids, and polybrominated diphenyl ethers (PBDEs) [18]. Looking at the chemical profiles in *L. herbacea*, it has been well accepted that this species has at least two chemotypes. One chemotype contains polychlorinated amino acids and sesquiterpenes, and the second chemotype only contains a mix of PBDEs, which can reach concentrations of up to 2% of the sponge dry mass [18,19]. For the latter chemotype, in 1993, Unson and co-workers discovered that PBDEs are mainly produced by its symbiotic bacteria, *Oscillatoria spongeliae* [18]. PBDEs are the major metabolites in *L. herbacea*, which have been shown in specimens that have been collected in several biogeographic regions [20,21,22]. There are also several reports of *L. herbacea* within the Indonesian Archipelago [23,24,25,26,27], which have also identified various bioactivities, including antibacterial [27,28,29,30], cytotoxicity [28,31], icthyotoxicity [29], antiproliferative activity [32], and protein inhibitor [33,34] activities. Reported bioactivities from this species have also been summarized in recent review articles [13,14,15].

However, while pharmacological activities have been well documented for extracts and certain purified compounds, little is known about their ecological function. Our study found multifaceted clinically relevant bioactivities in the crude extract and the isolated PBDEs. In addition, we were also able to identify an ecological function of the crude extract and its fractions by testing these crude extracts against environmental bacteria isolated from similar coral reef habitats as well as by demonstrating antifeeding activity for the isolated PBDEs, 3,4,5-tribromo-2-(2′,4′-dibromophenoxy)phenol (**1d**) and 3,4,5,6-tetrabromo-2-(2′,4′-dibromophenoxy)phenol (**2b**). To the best of our knowledge, only the tetra brominated diphenyl ether (BDE) 3,5-dibromo-2-(2′,4′-dibromophenoxy)phenol has been shown to exhibit antifeedant activity against fishes [19]. However, no ecological study on the activity of penta- and hexa-BDEs has been reported up until this point. Thus, the information provided here also fills an important gap by considering the ecological significance of PBDE derived from the marine sponge *L. herbacea*.

## 2. Results

### 2.1. Feeding Deterrent Assay

**Figure 1:**
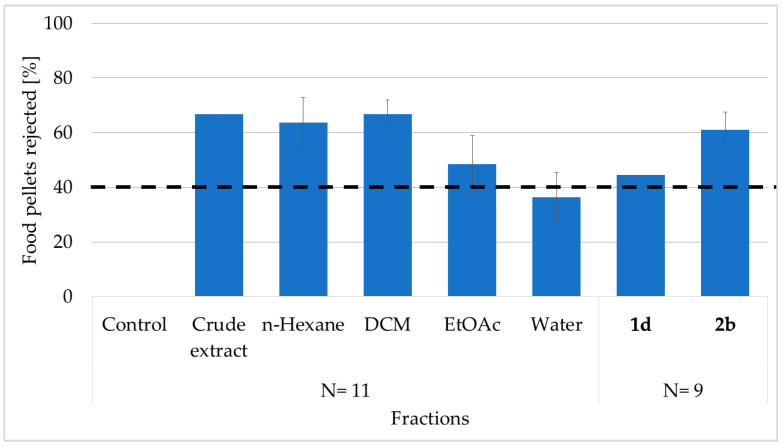
Feeding deterrence activity (%) of the crude extract, fractions (HEX, DCM, EtOAc, and H_2_O), and pure PBDEs (**1d** and **2b**) of *L. herbacea* against the pufferfish *C. solandri* (N = number of specimens tested in the assays). Bars represent the percent of rejected food pellets by the pufferfish (mean ± SD). The dashed line at 40% refers to significance in deterrence *p* < 0.05 (Fisher’s exact test). Control pellets contained no extract (only MeOH).

Crude extract of the marine sponge *L. herbacea* was tested in a feeding deterrent assay using the generalist fish predator, *C. solandri*. In total, 66.7% of the treated pellets containing crude extract were rejected by the pufferfish. Furthermore, a similar percentage of food pellets containing the n-hexane (HEX) or dichloromethane (DCM) fractions at natural volumetric concentrations also deterred feeding (63.6% and 66.7%, respectively). Since the ethyl acetate (EtOAc) and water (H_2_O) factions showed only low feeding deterrence with 48.5% and 36.4% percentages, the deterrent compounds had to be present in the HEX and DCM fractions (Figure 1).

We further isolated the major bioactive compounds by reverse-phase SPE fractionation followed by C_18_ reverse phased high-performance liquid chromatography (HPLC). HPLC purification of the active fractions yielded compounds **1d** and **2b** at levels of 2.9 mg and 6.8 mg, respectively. Pure compounds were injected at different concentrations to create a calibration curve, which was then used to determine the natural concentrations of the compounds in the sponge tissue. The analysis determined compounds **1d** and **2b** with concentrations of 1.6 mg and 2.4 mg, respectively, per mL sponge tissue. When tested at these respective concentrations, both PBDEs showed a significant deterrence at natural volumetric concentrations against the tested pufferfish. Compound **2b** displayed a high deterrence, with 61.1% of the pellets being rejected (*p* = 0.008, Fisher’s exact test), while compound **1d** revealed a somewhat lower but still significant deterrence, with 44.4% of the pellets being rejected (*p* = 0.041, Fisher’s exact test). The observed deterrence of the pure compounds was somewhat lower compared to the tested fractions or crude extract, except for the EtOAc and the water fraction, which were not significantly deterred (*p* = 0.32, Fisher’s exact test).

### 2.2. Antimicrobial Activity against Pathogenic and Non-Pathogenic Environmental Bacteria

**Figure 2:**
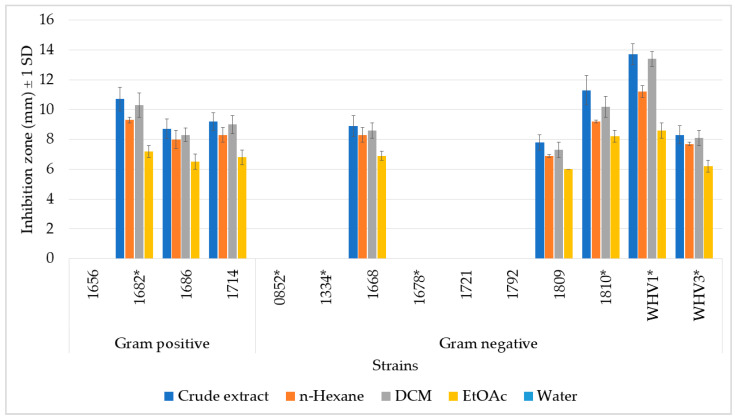
Total antibacterial activities of the crude extract and its organic fractions of the marine sponge *L. herbacea*. Y-axis shows inhibition zones in mm ± 1 SD for each tested strain. Gram-positive strains: 1656: *Streptomyces flavoviridis*; 1682 *: *Rhodococcus corynebacterioides*; 1686: *Exiguobacterium profundum*; 1714: *Mycobacterium franklinii*. Gram-negative strains: 0852 *: *Acitenobacter soli*; 1334 *: *Aliagarivorans marinus*; 1668: *Ruegeria areniliotoris*; 1678 *: *Acinetobacter calcoaceticus*; 1721: *Microbulbifer variabilis*; 1792: *Pseudovibrio denitrificans*; 1809: *Ruegeria areniliticus*; 1810 *: *Pantoea eucrina*; WHV1 *: *Aurantimonas coralicida*, WHV3 *: *Vibrio mediterranei*. (*): potentially pathogenic environmental bacteria.

An agar diffusion assay was used to determine possible antimicrobial activity against environmental bacteria, using 14 different pathogenic and non-pathogenic environmental bacteria, which had previously been isolated from a reef flat habitat (Figure 2) [35,36]. The extracts exhibited pronounced antimicrobial activities by inhibiting more than half of the test bacteria, of which four of them were putatively pathogenic. According to the Gram type of the bacteria, the crude extract and liquid-liquid separated fractions inhibited all of the Gram-positive bacteria except *Streptomyces* sp. (1656), while half of the Gram-negative bacteria were inhibited. Interestingly, the Agar diffusion assay (ADA) showed the same pattern as the one we found in the feeding assay. That is, the crude extract showed the highest activity, followed by its most lipophilic fractions, HEX and DCM. No inhibition zone appeared with the H_2_O fractions, while only weak inhibition occurred with the EtOAc fraction. All of the tested extracts and fractions showed the highest inhibition activity against WHV1 (*Aurantimonas coralicida*). Due to the lack of purified PBDEs, no further ADA experiments were performed.

### 2.3. Antimicrobial Assay against Pathogenic Clinical Microbes

The crude extract and fractions also displayed remarkable bioactivities in the antimicrobial assay against clinically relevant, pathogenic microbial, and fungal strains (cf. Table 1). The crude extract strongly inhibited *Bacillus subtilis*, *Staphylococcus aureus*, *Mucor hiemalis*, and *Rhodotorula glutinis*, whereas its activities against *Escherichia coli* and *Mycobacterium smegmatis* were weak. No activities were detected against *Pseudomonas aeruginosa* and *Candida albicans*. Pronounced activities were observed against all of the tested microbes with the HEX and DCM fractions. The EtOAc fraction only showed the potent inhibition of *B. subtilis* and moderate inhibition against *S. aureus,* *R. glutinis*, and *M. hiemalis*. Screening of the two purified compounds, **1d** and **2b**, also exhibited a broad range of antimicrobial activities against the seven test strains. The compounds were highly active against all tested strains except *E. coli* (MIC > 66.7 µg/mL). Both compounds strongly inhibited *B. subtilis* (MIC 0.5 μg/mL), displaying a five times lower MIC than the positive control tetracycline (2.8 μg/mL). Furthermore, compound **1d** exhibited bioactivity against the Gram-positive bacteria *S. aureus* that was as potent as the positive control gentamicin (MIC 0.5 μg/mL), whereas compound **2b** showed four times less bioactivity. Both compounds **1d** and **2b** were significantly more active (MIC 4.2 µg/mL and 2.1 µg/mL, respectively) than the positive control nystatin against the aerobic yeast cell *R. glutinis*.

### 2.4. Inhibitory Effects on HCV Infectivity

**Figure 3:**
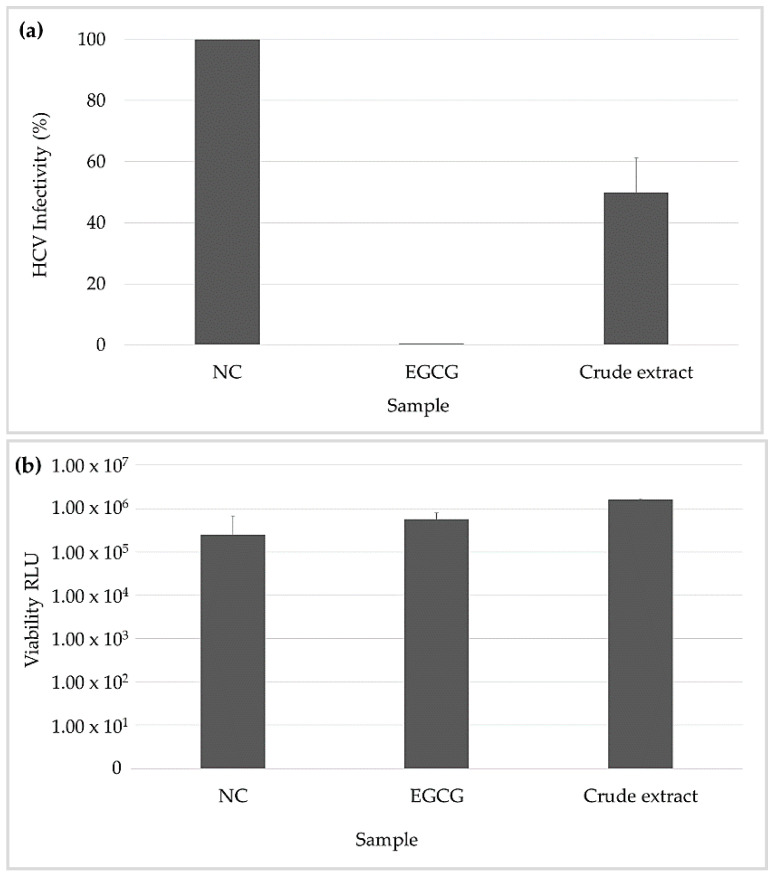
(**a**) shows the inhibition of infectivity by the Hepatitis C Virus (HCV). (**b**) illustrates the viability of Huh7.5 cells after the exposure of the tested crude extract. Bars represent the inhibition including the standard deviation (SD). MeOH was used as a negative control (NC), and epigallocatechin gallate (EGCG) was used as a positive control.

Next, we investigated the inhibitory effect of the *L. herbacea* crude extract against HCV infectivity in human liver cells. As depicted in Figure 3, only moderate inhibition of 50% was observed in contrast to the EGCG positive control. Therefore, we did not investigate its liquid-liquid fractions further. Interestingly, we found that the organic extract was not toxic in human liver cells, which was in contrast to previous studies that had reported cytotoxic effects by OH-PBDE compounds [30,37,38].

### 2.5. Structure Elucidation of Compounds ***1d*** and ***2b***

As the first step in structure elucidation, the exact masses of the purified compounds were obtained with a UPLC-HRMS (Waters Synapt G2-Si, Milford, MA, USA). Mass spectrometry (Waters Synapt G2-Si, Milford, MA, USA) was conducted in negative ionization mode. The HRESIMS of compound **1d** showed a pseudomolecular ion peak [M − H]^−^ = 574.6137 indicating the molecular formula of C_12_H_5_O_2_Br_5_. For compound **2b** an exact mass of [M − H]^−^ = 652.5246 was obtained (C_12_H_4_O_2_Br_6_). Both compounds were compared to compounds in the MarinLit database [39]. The results showed that both sum formulas were related to known PBDEs (Table 2).

**Figure 4:**
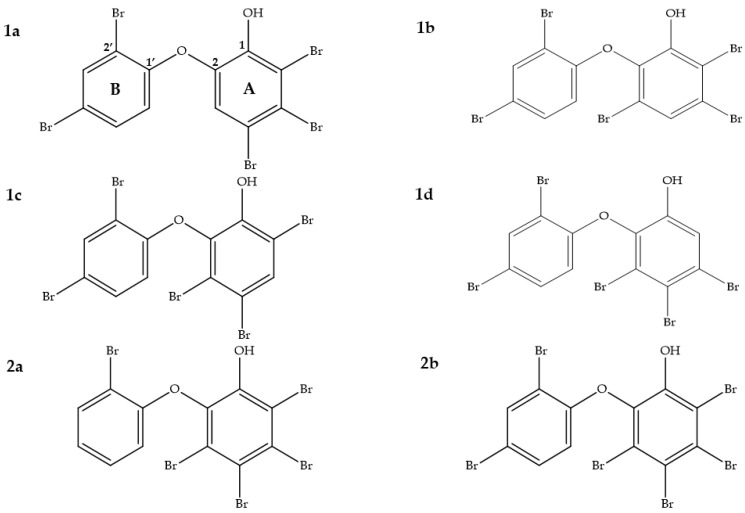
Four constitutional isomers of PBDEs are given to evaluate their ^13^C NMR chemical shift deviation (**1a**)–(**1d**) and to compare them to compound **1d** from our work. Penta-BDE, 3,4,5,6-tetrabromo-2-(2′-bromophenoxy)phenol (**2a**) that was first reported by Salva and Faulkner [42] is shown as a comparison to compound **2b** in our study.

To locate the positions of the bromine atoms in the ring system of compounds **1d** and **2b**, we conducted ^1^H and ^13^C NMR measurements. The results revealed that compound **1d** possessed three bromine atoms in the A-ring and two bromine atoms in the B-ring (Figure 4). In addition, an extra bromine atom in the A-ring was determined for compound **2b**.

The NMR data clearly indicated that the B-ring in both compounds **1d** and **2b** was identical. Since the A-ring is hexa-substituted in **2b**, there are no structural alternatives, whereas for **1d**, four constitutional isomers were possible (**1a** to **1d**). In order to elucidate the correct regioisomers, the ^1^H and ^13^C NMR data were compared to existing NMR data in the literature (Table 3 and Table 4). Furthermore, the structures of **1d** and **2b** were also verified through comparison with predicted ^13^C NMR chemical shifts [43,44,45,46] using three different computational approaches: NMRPredict [47], NMRShiftDB [48], and CSEARCH [49].

**Figure 5:**
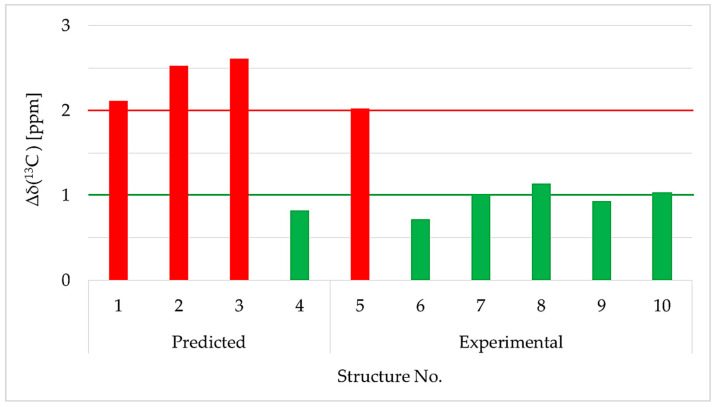
Comparison of the averaged ^13^C chemical shift deviations of the experimental to predicted values of constitutional isomers **1a** to **1d** (bars 1 to 4) and experimental data from the literature (bars 5 to 10). Bars 5 and 6 represent the two constitutional isomers described by Bowden et al., 2000 [50], which are identical to **1b** and **1d** in the current work. Green bars indicate deviations of 1 ppm or less (exception bars 8 and 10), whereas red bars indicate 2 ppm or higher deviations. Compound numbers were mentioned in the original publication: (5) Bowden et al., 2000 [50]—compound No. 1; (6) Bowden et al., 2000 [50]—compound No. 2 and Agarwal and Bowden 2005 [52] compound No. 3; (7) Fu and Schmitz 1996 [53]—compound No. 5; (8) Pedradab 2005 [54] compound No. 12; (9) Fu et al., 1995 [21]—compound No. 13; (10) Utkina et al., 2019 [55]—compound No. 2 (DMSO-*d*_6_).

The averaged deviation of δ(^13^C) for all 12 carbon atoms to the calculated and existing experimental data sets in comparison to our experimental values is given in Figure 5. The results showed that the mean value of **1d** was close to the experimental shifts reported for **1d** in the literature [49] (Figure 5, bars 6 to 10). Comparison to the calculated values obtained with NMRPredict also preferred constitutional proposals **1d** (<1 ppm) over the regioisomers **1a** to **1c** (>2 ppm). Therefore, **1d** was confirmed as 3,4,5-tribromo-2-(2′,4′-dibromophenoxy)phenol (Figure 4). In fact, our δ(^13^C) values for compound **1d** were 100% identical to the one’s published by Suyama et al. [40] (Table 4, not shown in Figure 5).

Compound **2b** showed very similar ^13^C chemical shifts compared to the only reported NMR data from Utkina et al. [41] (Table 5 and Table 6). Since we do not have constitutional isomers for the A-ring in compound **2b**, we decided to use another compound with the same A-ring in order to determine how well the ^13^C chemical shifts can be predicted for that ring. A similar compound, 3,4,5,6-tetrabromo-2-(2′-bromophenoxy)phenol (**2a**), which was first identified by Salva and Faulkner [50], with one less bromine in the B-ring compared to compound **2b** was used for comparison (Figure 4). The ^13^C chemical shifts of the A-ring (C-1 to C-6) of both compounds showed high similarity (Table 6). Therefore, compound **2b** was confirmed as 3,4,5,6-tetrabromo-2-(2′,4′-dibromophenoxy)phenol (Figure 4).

## 3. Discussion

This is the first study to demonstrate the bioactivity of crude extracts, fractions, and pure compounds in ecological assays for the Indonesian marine sponge *L. herbacea*. Two of the major metabolites responsible for the observed bioactivities were successfully isolated and confirmed by UPLC-HRMS and NMR structure elucidation. Both compounds were known PBDEs, namely 3,4,5-tribromo-2-(2′,4′-dibromophenoxy)phenol (**1d**) and 3,4,5,6-tetrabromo-2-(2′,4′-dibromophenoxy)phenol (**2b**).

Compound **1d** was first characterized and described in 1969 from *Dysidea herbacea* from Palau, Micronesia, using only proton NMR spectral data. In the following years, the structure was verified by partial synthesis and further proton NMR analysis [20,51]. Both compounds were also the major metabolites in a different specimen of *Dysidea* [20,21]. Ten years later, the ^13^C NMR data of compound **1d** were reported for the first time along with new bioactivities, such as the inhibition of inosine monophosphate dehydrogenase (IMPDH), guanosine monophosphate synthetase, and 15-lipoxygenase [21]. Later, compound **1d** was detected together with novel PBDEs [50,52,53]. In 2008, Ortlepp and colleagues also reported the antifouling activity of compound **1d** against several fouling organisms, such as the axenic diatom *Amphora coffeaeformis*, the blue mussel *Mytilus edulis* as well as antibacterial activities isolated from marine biofilms [56]. However, compound **1d** was considered to be a toxic compound to the barnacle cyprids in these assays. Suyama and his co-workers rediscovered compound **1d** in a red algae cyanobacteria mix (*Leptofaucia* sp. and *Oscillatoria* sp., respectively) from the Grabo Reef in Papua New Guinea [40]. This is not surprising, as Unson and colleagues had previously demonstrated that the 3,5-dibromo-2-(2′,4′-dibromophenoxy)phenol reported from *Dysidea herabcea* was not produced by the sponge itself but rather by its cyanobacterial symbiont (filamentous cyanobacterium), which closely resembles *Oscillatoria spongeliae* [18], indicating that cyanobacteria, especially of the genus *Oscillatoria*, is one of the main sources of PBDEs in nature. Suyama and colleagues also provided a few corrections on the ^13^C chemical shift data that closely match our results using the same CDCl_3_ solvent [40].

Compound **2b,** on the other hand, was first discovered in the 1980s from the marine sponge *Dysidea fragilis* using HRMS and NMR analysis [41]. The compound exhibited antimicrobial activity against the clinically relevant Gram-positive bacteria *S. aureus* and *B. subtilis* [51]. The compound was reisolated recently with the same spectroscopic data along with compound **1d**. The study revealed that adding more bromine positions in the B-ring increased the inhibitory potency for the α-D-galactosidase from the marine bacterium *Pseudoalteromonas* sp. KMM 701 of the GH36 family [55]. Furthermore, the multifaceted activities of compound **2b** were also reported by Handayani et al. [30]. The compound showed potent antibacterial activity against the human pathogenic bacteria *B. subtilis* with a MIC of 3.1 µg/mL along with high cytotoxicity in the brine shrimp lethality test (BSLT), with an LC_50_ of 0.9 µg/mL. Antifungal activity was shown against *Cladosporium cucumerinum* at 50 nmol (8 mm inhibition zone) and 25 nmol (5 mm inhibition zone). Antiproliferative activity of compound **2b** has been reported against MCF-7 human adenocarcinoma breast cancer cells [37]. Unfortunately, NMR chemical data on compound **2b** has only ever been incompletely provided in the more recent literature described above.

Information on deterrent activities of extracts from the sponge *Dysidea* started in the 1990s when Duffy and Paul reported deterrent activities against fishes during field feeding assays with tetra-brominated BDEs from *Dysidea* sp. collected in Guam, Micronesia [19]. Unfortunately, only the structure of the BDE was reported, and no structural data were reported for the compound, as they instead referred to the original description of the compound 3,5-dibromo-2-(2′,4′dibromophenoxy)phenol, which Carte and Faulkner [20] described. The compound deterred the fishes in two kinds of feeding assay (paired and multiple-choice assay), where the incorporation of BDE in food pellets resulted in 40–70% rejection rates [19]. In a follow-up study by Pennings et al. [57], the deterrent activity of the compound was again confirmed with general reef fish and the pufferfish *C. solandri* and the crab *Leptodius* spp. in aquarium and field assays at 1, 2, and 4% dry mass. Faulkner et al. also confirmed an ecological role of observed BDEs in *Dysidea herbacea* from Palau, Micronesia. BDEs were abundantly located in needle-like forms and feather-like patterns in the ectosomal layer of the sponge [58]. They conclude that the accumulation of BDEs in the ectosomal layer of the sponge coupled with the feeding deterrent and antimicrobial activities indicates an ecological role of these compounds in defense against bacteria, crab, and fish predators. Further evidence for the defensive role of BDEs was provided by Becerro and colleagues [59], who detected 3,5-dibromo-2-(2′,4′-dibromophenoxy)phenol in the sponge *Dysidea granulosa* from Guam and the sponge feeding gastropterids *Sagaminopteron nigropunctatum* and *S. psychedelicum*. Quantification of the compound revealed its accumulation by both sponge predators in the gastropterids tissue at over twice the sponge concentration, most likely for defense against fish predators [59].

Our results are the first report of sponge-derived PBDEs, penta- and hexa- brominated diphenyl esters (compounds **1d** and **2b**), exhibiting significant deterrence in the fish feeding assay and in antimicrobial activities against ecological relevant microorganisms. We also found that the presence of one additional bromine on the phenolic A-ring resulted in a higher fish deterrence but not in antimicrobial activity. However, since PBDEs are the major metabolites in the crude extracts (Figure S3) and have considerable antibacterial and antifungal activity against clinically relevant microorganisms, one can assume that PBDEs are also responsible for the observed activities against environmental bacteria. Furthermore, they likely serve an important ecological function, as they were especially active against environmental pathogenic strains, such as *Vibrio mediterranei* and *Aurantimonas coralicida*, which have been implicated in several diseases of marine organisms [60,61]. In addition, pathogenic bacteria *Pantoea eucrina* and *Rhodococcus corynebacterioides* infections have been reported in humans [62,63].

The pronounced bioactivity of the two isolated PBDEs was further confirmed in antimicrobial assays with clinically relevant strains, where both compounds exhibited high inhibitory activity against *B. subtilis*, that was even five times stronger than the positive control tetracycline (MIC 2.8 µg/mL). Furthermore, compound **1d** also inhibited *S. aureus* at the same concentration as the control, while it showed weak activity against *E. coli* and *A. baumannii.* In addition, both compounds also exhibited pronounced antifungal activity against *R. glutinis,* demonstrating MIC values that were around ten times lower (4.2 µg/mL and 2.1 µg/mL, respectively) than those of the positive control nystatin (33.3 µg/mL). The yeast *Rhodoturula* is a ubiquitous genus in different ecosystems, colonizing plants, humans, and other mammals [64]. *R. glutinis* was also found in benthic animals and deep-sea sediments from the northwest Pacific Ocean [65]. The pathogenicity of the genus *Rhodoturula* towards animals and humans by causing bloodstream infections, short bowel disease, and acute lymphoid leukemia has been reviewed comprehensively [64]. The emerging resistance of *R. glutinis* and other *Rhodoturula* species against caspofungin (100%), fluconazole (94.7%), luliconazole (1–8 µg/mL), and voriconazole (74.4%) has been well documented [66] and emphasizes the need for potent antifungal compounds such as **1d** and **2b**. Both compounds seem to have broad antifungal activity, as seen by their pronounced inhibition of *M. hiemalis.* Compound **1d** also exhibited about 30% stronger activity against *P. anomala* than the positive control nystatin. Moderate fungicidal activity was also reported for compound **2b** against *C. cucumerinum* [30].

*L. herbacea* crude extract showed activity in the HCV inhibitory assay. However, inhibition was weak compared to the positive control epigallocatechin gallate (EGCG) and therefore was not followed up upon with pure compounds. These results differed from a previous study that showed novel HCV inhibitory activity from the PBDE 6-hydroxy-2,2′,4,4′-tetrabromodiphenyl ether from a Japan *Dysidea* sp [38].

Based on the bioactivity results reported here and previous literature reports, one can assume that PBDEs have an important ecological function in protecting organisms against various fish predators and pathogenic microorganisms. The production of these defensive metabolites might be one reason why *L. herbacea* is one of the dominant benthic invertebrates in certain areas [67]. In Indonesia, the dominance of *Dysidea* was recently recorded in the Wakatobi Marine National Park, Sulawesi, particularly in areas with high sedimentation [24,68]. Further research towards the ecological roles of PBDEs and especially their role in competitions with other sessile benthic invertebrates could close the gap of why *Lamellodysidea* is the prevalent inhabitant of shallow-water coral reefs throughout the Indo-Pacific.

## 4. Materials and Methods

### 4.1. Sample Collection

*Lamellodysideaherbacea* was collected by hand in Pari Island, Kepulauan Seribu, Indonesia, in August 2017. *L. herbacea* were taken at 3–8 m depths. The samples were cut with a scalpel and were then transferred into an ambient seawater-filled plastic bag. *L. herbacea* was morphologically identified as belonging to the Dysideidae family, Dictyoceratida order, and Demospongiae class. Collected samples were transferred to the nearest lab, frozen, and then transported to the Environmental Biochemistry working group, Carl von Ossietzky Universität Oldenburg, Oldenburg, Germany.

### 4.2. Extraction and Isolation

A wet weight sample of the sponge (243 g) was freeze-dried (31.65 g), grounded into powder, and extracted exhaustively with MeOH: EtOAc (1:1; HPLC grade VWR International GmbH, Darmstadt, Germany). Bioassay-guided isolation was conducted using 558 mg of the total organic extracts (4.1 g), which were further polarity partitioned into HEX (96.6 mg), DCM (127.8 mg), EtOAc (8.1 mg), and H_2_O (305.9 mg). The HEX and DCM fractions were chosen to be tested for all bioactivity screening assays except the HCV inhibitory assay. For identification of the potential bioactive compounds fractions were further separated using an HPLC (Agilent) fraction collector coupled with a UPLC-HRMS (MaXis ESI TOF, Bruker Daltonik GmbH, Bremen, Germany) with a BEH C_18_ column (Waters ACQUITY, Milford, MA, USA) (1.7 µm 2.1 × 50 mm) [63]. A step gradient was used from 95% H_2_O and 5% MeCN (solvent A) to 5% H_2_O and 95% MeCN (solvent B). The resulting fractions were collected in a microplate and tested in MIC assays with clinically relevant pathogenic bacteria (see 4.5 for details). MS and MS/MS of the active peaks were obtained using UPLC-HRMS (Waters Synapt G2-Si, Milford, MA, USA) with the same gradient and column system described above and the MassLynx 4.2 data analysis program.

The masses of the MS of the targeted compounds were obtained in the negative mode. Detected masses were compared to various databases, namely MarinLit (Royal Society of Chemistry, Cambridge, UK), Dictionary of Natural Product (ChemNetBase, Taylor&Francis, Abingdon, UK), and Google scholar. We identified the two targeted compounds as known compounds, namely the 3,4,5-tribromo-2-(2′,4′-dibromophenoxy)phenol (**1d**) and 3,4,5,6-tetrabromo-2-(2′,4′-dibromophenoxy)phenol (**2b**). Solid-Phased Extraction C_18_-based (SPE) fractionation was applied to each of the HEX and DCM fractions (used 73 mg each) to isolate the targeted compounds using a C_18_ reversed-phase cartridge (10 g) with a column capacity of 75 mL (HyperSep C_18_, Fischer Scientific, Leicestershire, UK). The major OH-BDE compounds were in the fraction of 70:30 CH_3_CN: H_2_O (38.6mg).

Purification of the SPE fraction was performed by high-performance liquid chromatography (HPLC) (Agilent 1260 Infinity, Agilent, Santa Clara, SA, USA) using a reversed-phase C_18_ (Pursuit XRs 5 µm C_18_ 250 × 10.0 mm, Agilent, Santa Clara, SA, USA) semi-preparative column. CH_3_CN and H_2_O with the addition of formic acid (FA, 98%, Carl Roth, Karlsruhe, Germany) at a concentration of 0.1% to both the solvent were applied in the running sequence. The column temperature was set at 40 °C. A flow rate of 2 mL/min was applied using a step gradient (sequence: in 50 min 85:15 CH_3_CN: H_2_O to 95:5 CH_3_CN: H_2_O, 2 min 95:5 CH_3_CN: H_2_O to 100:0 CH_3_CN: H_2_O, 33 min 100:0 CH_3_CN: H_2_O, 2 min 100:0 CH_3_CN: H_2_O to 85:15 CH_3_CN: H_2_O then 13 min 85:15 CH_3_CN: H_2_O). Compounds were detected at 280 nm with the Diode Array Detector (DAD; Agilent 1260 Infinity Diode Array Detector (G4212-60008), Agilent, Santa Clara, SA, USA). The target compounds were eluted between 14 and 16 min. Purification yielded 3,4,5-tribromo-2-(2′,4′-dibromophenoxy)phenol (**1d**) (2.9 mg) and 3,4,5,6-tetrabromo-2-(2′,4′-dibromophenoxy)phenol (**2b**) (6.8 mg). The NMR spectra were measured on a Bruker 700 MHz cryo NMR spectrometer (Avance III HD) using Topspin 3.6.2 for analysis. The structures of compounds **1d** and **2b** were verified with the following NMR experiments: 1D proton, ^1^H,^13^C-HSQC (pulse program: hsqcedetgpsisp2.3), and ^1^H,^13^C-HMBC (pulse program: hmbcgplpndqf) [50,51,52]. The ^13^C NMR chemical shifts were calculated with the programs NMRPredict, NMRShiftDB, and CSEARCH. However, only the results of the first method are given in the main manuscript (Table 4). The results of the other two methods are provided in the Supplementary Materials (Table S1).

The compound 3,4,5-tribromo-2-(2′,4′-dibromophenoxy)phenol (**1d**): white powder; molecular formula C_12_H_4_Br_5_O_2_^−^; UV (MeOH, photodiode array), λmax 290.407 nm; HRMS-ESI *m/z* [M − H]^−^: 574.614, 576.612, 578.610, 580.607, 582.606, 584.604. The ^1^H and ^13^C NMR data of the structure are in Table 3 and Table 4.

The compound 3,4,5,6-tetrabromo-2-(2′,4′-dibromophenoxy)phenol (**2b**): white crystal powder; molecular formula C_12_H_3_Br_6_O_2_; UV (MeOH, photodiode array), λmax 281.407 nm; HRMS-ESI *m/z* [M − H]^−^: 652.524, 654.521, 656.519, 658.518, 660.516, 662.513, 664.512. The ^1^H and ^13^C NMR data of the structure are in Table 5 and Table 6.

### 4.3. Feeding Deterrence

Feeding deterrence assays were conducted using a modified method from Pawlik [69], according to Helber et al. [70]. Thirteen pufferfish, *Canthigaster solandri* (Richardson, 1845), were chosen as model organisms since they are omnivorous invertebrate predators known to feed on sponges and since they have been used in several previous studies [35,70,71]. The assay was conducted using two kinds of artificial food pastes (0.05 g alginic, 0.075 g powdered squid mantle, one drop food color) consisting of treatment food with natural volumetric concentration of sponge crude extract, fractions or pure compounds added and without sponge chemistry as a control. The paste was made by mixing the ingredients with distilled water that were each loaded into separate 2 mL syringes. A long strand form was created by emptying the syringe content onto the plates containing a 0.25 M calcium chloride solution. The strand was rinsed with seawater several times and was then cut into small pellets to ensure that the puffers could eat them whole.

Thirteen individual puffers were maintained by feeding them with the pellets without the extract for several days before the assays. The assay was started by offering a control and treatment pellet sequentially to each puffer. The puffers were not scored if they did not eat the first control pellet. If the control was eaten, the treated pellet was then offered, and rejection was scored if the fish spat the pellet out or mothed the pellets (see previous studies for details) [70]. Next, another control pellet was offered to confirm that the fish was still hungry. The rejection was not scored if the second control was ignored or rejected. The pellet rejection of the first and second control was excluded from the calculation. The assay was continuously conducted until at least ten puffers participated in the assay. Fisher’s exact test was applied for validating significant differences in the palatability between the control and treated pellet [70]. Further feeding assay with the two purified compounds were also conducted testing them at their natural concentration. The concentration was obtained by calculating the compounds’ total peak area and by comparing it to a calibration curve (Figure S2).

### 4.4. Agar Diffusion Assay (ADA) against Pathogenic and Non-Pathogenic Environmental Bacteria

Crude extracts were tested against 14 marine environmental bacteria strains, including pathogenic and non-pathogenic bacteria associated with marine diseases, using a modified version of the disk diffusion method [71]. The strains were identified and used by our colleagues in similar assays [35,72]. Marine broth (MB) agar plates were inoculated with 200 μL of the test strain cultures (24 h cultured in 10 mL liquid broth medium). Subsequently, different crude extracts (conc. 0.5 mg/disk), a negative control (MeOH: EtOAc, 15 μL/disk), and positive control (0.1 M Chloramphenicol, Oxoid^®^, Z 15 μL/disk) were pipetted onto sterile paper disks (Ø 6 mm, Whatman) and dried. The impregnated disks were applied upside down onto the agar medium plates at equidistant points. The diameters of inhibition zones were measured to the closest mm after incubation at 37 °C for 24 and 48 h. The assays were conducted in three replicates. The results were expressed by calculating the average of the total inhibition zone surrounding the disks with the crude extract or fractions.

### 4.5. Minimum Inhibition Concentration (MIC) Antimicrobial Assay

To evaluate the bioactivity of the fractions against pathogenic bacteria and fungi, a minimum inhibition concentration (MIC) assay in 96-well microplates was conducted following Okanya et al. [73]. The samples were tested using a panel consisting of Gram-positive Bacteria *Bacillus subtilis* (DSM 10), *Staphylococcus aureus* (DSM 346), and Gram-negative bacteria *Escherichia coli* (DSM 1116), *Pseudomonas aeruginosa* (PA14), and Mycobacterium smegmatis (ATCC700084). Additional antifungal assays against the filamentous fungus, *Mucor hiemalis* (DSM 2656), and the yeasts *Candida albicans* (DSM 1665) and *Rhodotorula glutinis* (DSM 10134) were conducted. The bacterial strains were added into 20 mL MHB (Mueller Hinton Broth) medium, except for *Mycobacterium smegmatis*, which was added into Middlebrook 7H9 medium. Yeast and fungal strains were added into 20 mL MYC medium (1.0% phytone peptone, 1.0% glucose, 50 mM HEPES (11.9 g/L) pH 7.0). Assay starting conditions were adjusted to OD600 of 0.01 for the bacteria and to 0.05 for the yeast/fungi.

The MIC test was conducted by first pipetting 20 μL aliquots (10 mg/mL) of the sample into 280 μL of microbial culture in the well of the first row (A). Then, serial dilution steps (1:2) were applied until the eighth row (H). The result was evaluated with visual observation after overnight incubation (for *Mycobacterium smegmatis* was 48 h) on a microplate-vibrating shaker (Heidolph Titramax 1000, Heidolph Instruments GmbH & Co.KG, Schwabach, Germany) at 600 rpm and at 30 °C (for *E. coli*, *P. aeruginosa*, *M. smegmatis* was 37 °C). Negative and positive controls were also applied (Table 1). Clear wells indicated no growth. Compounds were considered more potent if higher dilutions still resulted in no growth (the most potent extracts still inhibited test strains in the eighth row (H)).

Fractions were collected every 30 s using an Agilent 1100 HPLC system equipped with a fraction collector. The fractions were collected in 96-well plates and were dried [74], followed by inoculation with the test bacteria. The growth inhibition in the wells was correlated with the chromatogram peak based on the retention time. UPLC-HRMS was further used to analyze the mass and the UV spectrum of the active target compounds.

### 4.6. Inhibitory Effects on HCV Infectivity

Huh7.5 cells, steadily expressing *Firefly* luciferase (Huh7.5 Fluc; 1 × 10^4^/well) were provided by Charles Rice (Rockefeller University, New York, NY, USA) which were generated by the stable retroviral transduction of the gene encoding for the *Firefly* luciferase (FLuc) via a transduction approach described elsewhere [75]. The cells were provided by Charles Rice (Rockefeller University, New York, NY, USA). Those were seeded in 96-well plates with Dulbecco’s modified Eagle’s medium (DMEM, Gibco, Thermo Fisher Scientific, Schwerte, Germany) containing 2 mM glutamine, 1% minimum essential medium nonessential amino acids (MEM NEAA, Gibco, Thermo Fisher Scientific, Schwerte, Germany), 100 µg/mL streptomycin, 100 IU/mL penicillin (Gibco, Thermo Fisher Scientific, Schwerte, Germany), 10% fetal bovine serum, and 5 µg/mL blasticidin. Cells were incubated at 37 °C with a 5% CO_2_ supply. The assay was conducted by infecting Huh7.5 cells with the Jc1-derived *Renilla* reporter viruses in the presence or absence of sponge crude extracts as described elsewhere [72]. Reporter virus infection was determined by *Renilla* luciferase activity. The cell viability was measured through the determination of *Firefly* luciferase, which was stably expressed in the target cells. Epigallocatechin gallate (EGCG; was purchased from Sigma-Aldrich, Seelze, Germany) was a positive control. After three days of incubation, the infected cells were washed with PBS, and 50µL H_2_O was added afterward. The infected cells were lysed and then frozen at −80 °C for 1 h following Renilla and Firefly luciferase activity measurements on a Berthold Technologies Centro XS3 Microplate Luminometer (Berthold Technologies GmbH & Co.KG, Bad Wildbad, Germany) as indicators of viral genome replication and cell viability, respectively.

## 5. Conclusions

The two isolated PBDEs (**1d**/**2b**) from the Indonesian marine sponge *L. herbacea* were characterized by detailed LC-MS and NMR experiments. Additionally, we used three different chemical shift calculation programs to verify the bromine positions within the PBDE 3,4,5-tribromo-2-(2′,4′-dibromophenoxy)phenol (**1d**). Both compounds showed remarkable antimicrobial and feeding deterrent activities. Their ecological role in defense against fish predators and microbial pathogens had so far not been reported and might be a reason for the widespread occurrence of this sponge in shallow water coral reef habitats throughout the Indo-Pacific. This study also provided additional detailed activity results against various clinical pathogenic bacteria and fungi, demonstrating the bioactivity of the investigated PBDEs as potential antimicrobial and antifungal agents.

## Figures and Tables

**Table 1:** The minimum inhibitory concentrations (MIC) of the crude extract, its fractions, and compounds **1d** and **2b** against different human pathogenic bacteria and fungi. If the activity of the crude extract was not pronounced (> 66.7 µg/mL) and if the fractions or pure compound amounts were limited, no further testing was conducted with the strain. Test compound activities were compared to the positive controls: gentamicin, tetracycline, and nystatin.

Sample	MIC [µg/mL]									
	Gram-Positive Strains			Gram-Negative Strains			Fungi			
	Bs	Sa	Ms	Ec	Pae	Ab	Rg	Mh	Ca	Pa
Crude Extract	0.5	0.5	66.7	66.7	>66.7	8.3	1.0	0.5	>66.7	2.1
HEX	0.5	0.5	n.t.	1.0	n.t.	2.1	0.5	0.5	n.t.	1.0
DCM	0.5	0.5	n.t.	1.0	n.t.	1.0	0.5	1.0	n.t.	0.5
EtOAc	1.0	4.2	n.t.	>66.7	n.t.	>66.7	16.7	16.7	n.t.	33.3
H_2_O	66.7	>66.7	n.t.	>66.7	n.t.	>66.7	n.t.	>66.7	n.t.	>66.7
**1d**	0.5	0.5	n.t.	>66.7	n.t.	16.7	4.2	8.3	n.t.	4.2
**2b**	0.5	2.1	n.t.	>66.7	n.t.	>66.7	2.1	8.3	n.t.	13.9
Gentamicin	n.t.	0.5	n.t.	0.5	n.t.	2.8	n.t.	n.t.	n.t.	n.t.
Tetracycline	2.8	n.t.	n.t.	n.t.	n.t.	n.t.	n.t.	n.t.	n.t.	n.t.
Nystatin	n.t.	n.t.	n.t.	n.t.	n.t.	n.t.	33.3	8.3	n.t.	5.6

Gram-positive strains: Bs: *Bacillus subtilis*; Sa: *Staphylococcus aureus*; Ms: *Mycobacterium smegmatis*. Gram-negative strains: Ec: *Escherichia coli*; Pae: *Pseudomonas aeruginosa*; Ab: *Acinetobacter baumannii*. Fungi: Rg: *Rhodotorula glutinis*; Mh: *Mucor hiemalis*; Ca: *Candida albicans*; Pa: *Pichia anomala*; other abbreviations: n.t.: not tested.

**Table 2:** MS data for the two isolated compounds **1d** and **2b**. The exact masses of both compounds were compared to the compounds in the MarinLit database (±0.005 Dalton).

Compound	Observed MS	Result in MarinLit	References
**1d**	574.6137 [M−H]^−^ 575.6210 [M]	3,4,5-tribromo-2-(2′,4′-dibromophenoxy)phenol [M] = 575.6210	[40]
**2b**	652.5246 [M−H]^−^ 653.5318 [M]	3,4,5,6-tetrabromo-2-(2′,4′-dibromophenoxy)phenol [M] = 653.5315	[41]

**Table 3:** Comparison of ^1^H NMR data of compound **1d** with related structures reported in previous studies.

Position	1d (This Work)	Literature Data										
		Sharma and Vig 1972 1d	Carte and Faulkner 1981 1d	Bowdenet al., 2000 1b	Bowdenet al., 2000 1d	Fu and Schmitz 1996 1d	Pedpradab 2005 1d	Fu et al., 1995 1d	Fu et al., 1995 1d	Fu et al., 1995 1d	Sumaya et al., 2010 1d	Utkina et al., 2019 1d
	δ(^1^H)	δ(^1^H)	δ(^1^H)	δ(^1^H)	δ(^1^H)	δ(^1^H)	δ(^1^H)	δ(^1^H)	δ(^1^H)	δ(^1^H)	δ(^1^H)	δ(^1^H)
1	OH	OH	OH	OH	OH	OH	OH	OH	OH	OH	OH	OH
2	OR	OR	OR	OR	OR	OR	OR	OR	OR	OR	OR	OR
3	Br	Br	Br	Br	Br	Br	Br	Br	Br	Br	Br	Br
4	Br	Br	Br	7.55	Br	Br	Br	Br	Br	Br	Br	Br
5	Br	Br	Br	Br	Br	Br	Br	Br	Br	Br	Br	Br
6	7.45	7.50	7.42	Br	7.42	7.43	7.41	7.42	7.51	7.38	7.44	7.45
1′	OH	OH	OH	OH	OH	OH	OH	OH	OH	OH	OH	OH
2′	Br	Br	Br	Br	Br	Br	Br	Br	Br	Br	Br	Br
3′	7.80	7.80	7.76	7.78	7.79	7.84	7.85	7.77	7.82	7.78	7.79	7.90
4′	Br	Br	Br	Br	Br	Br	Br	Br	Br	Br	Br	Br
5′	7.31	7.38	7.26	7.28	7.29	7.35	7.31	7.27	7.39	7.28	7.29	7.40
6′	6.42	6.56	6.41	6.41	6.41	6.51	7.85	6.38	6.63	6.42	6.41	6.51

The ^1^H chemical shifts of compound **1d** (this work, solvent CDCl_3_) were compared to literature data (the atom numbering of Bowden et al., 2000 [50] was used for all literature data). Compound source of the literature comparison (compound numbers and solvents used mentioned in the original publication): Sharma and Vig 1972 [51]—compound No. 1 (D_2_O); Carte and Faulkner 1981 [20]—compound No. 1 (CCl_4_); Bowden et al., 2000 [50]—compound No. 1(CDCl_3_); Bowden et al., 2000 [50]—compound No. 2 (CDCl_3_) also mentioned in Agarwal and Bowden 2005 [52]—compound No. 3 (CDCl_3_); Fu and Schmitz 1996 [53]—compound No. 5 (DMSO-*d*_6_); Pedradab 2005 [54]—compound No. 12 (DMSO-*d*_6_); Fu et al., 1995 [21]—compound No. 13 (CDCl_3_), (Acetone-*d*_6_), (DMSO-*d*_6_); Suyama et al., 2010 [40]—compound No. 1b (CDCl_3_); Utkina et al., 2019 [55]—compound No. 2 (DMSO-*d*_6_).

**Table 4:** ^13^C NMR data of compound **1d** compared to predicted values and literature data for other similar compounds.

Position	δ^13^C [ppm]											
	1d (This Work)	1a *	1b *	1c *	1d *	Bowden et al., 2000-1b	Bowden et al., 2000-1d	Fu and Schmitz 1996-1d	Pedpradab 2005-1d	Fu et al., 1995-1d	Sumaya et al., 2010-1d	Utkina et al., 2019-1d
1	148.9	150.2	148.3	146.7	151.5	148.1	148.9	150.8	151.4	150.9	148.9	150.8
2	139.5	143.8	138.7	140.9	139.5	138.7	139.9	139.5	139.7	139.7	139.5	139.4
3	121.1	122.1	116.8	120.2	121.4	116.7	113.6	121.5	121.9	121.6	121.1	121.6
4	119.2	115.5	128.0	116.1	116.1	128.0	119.3	115.9	116.2	116.1	119.2	116.0
5	122.9	121.8	122.1	132.8	121.7	122.2	122.9	121.5	122.2	121.7	122.9	121.6
6	120.7	116.5	113.3	109.8	121.0	113.4	120.8	120.7	121.9	120.9	120.7	120.5
1′	151.7	152.1	152.1	152.2	152.3	152.1	151.8	152.3	152.6	152.4	151.7	152.3
2′	112.7	115.0	112.6	112.4	111.9	112.6	112.8	111.7	111.9	111.9	112.7	111.8
3′	136.3	135.4	135.7	135.7	135.7	136.1	136.4	135.1	135.9	135.3	136.3	135.1
4′	116.2	116.1	116.1	116.1	116.1	115.8	116.3	114.1	114.5	116.0	116.2	114.0
5′	131.6	132.6	131.4	131.4	131.4	131.3	131.6	131.5	131.9	131.6	131.6	131.7
6′	115.7	120.8	121.9	116.9	115.8	115.8	115.8	115.9	114.5	114.4	115.7	115.9

The ^13^C chemical shifts of compound **1d** (this work) were compared to predicted values and literature data (the atom numbering of Bowden et al., 2000 [50] was used for all literature data). The columns with the calculated ^13^C chemical shift values (columns 3 to 6) using NMRPredict are indicated by an asterisk (*). The carbon atoms C-3 to C-6 were not explicitly assigned for the prediction of the constitutional isomers **1a** to **1c** due to very similar ^13^C chemical shifts (119.2 to 122.9 ppm). Columns 7 to 13 represent reported literature data. Compound source of the literature comparison (compound numbers and solvents used were mentioned in the original publication): Bowden et al., 2000 [50]—compound No. 1 (CDCl_3_); Bowden et al., 2000 [50]—compound No. 2 (CDCl_3_) also mentioned in Agarwal and Bowden 2005 [52]—compound No. 3 (CDCl_3_); Fu and Schmitz 1996 [53]—compound No. 5 (DMSO-*d*_6_); Pedradab 2005 [54]—compound No. 12 (DMSO-*d*_6_); Fu et al., 1995 [21]—compound No. 13 (DMSO-*d*_6_); Suyama et al., 2010 [40]—compound No. 1b (CDCl_3_); Utkina et al., 2019 [55]—compound No. 2 (DMSO-*d*_6_).

**Table 5:** Comparison of ^1^H NMR data of compound **2b** with related structures reported in previous studies.

Position	2b (This Work)	Literature Data	
		Utkina et al., 1987 2b	Salva and Faulkner 1990 2a
	δ(^1^H)	δ(^1^H)	δ(^1^H)
1	OH	OH	OH
2	OR	OR	OR
3	Br	Br	Br
4	Br	Br	Br
5	Br	Br	Br
6	Br	Br	Br
1′	OR	OR	OR
2′	Br	Br	Br
3′	7.80	7.79	6.52
4′	Br	Br	6.99
5′	7.30	7.29	7.18
6′	6.41	6.42	7.65

The ^1^H chemical shifts of compound **2b** (this work, solvent: CDCl_3_) were compared to literature data (the atom numbering of Salva and Faulkner [42] was used for all literature data). The compound was confirmed to be identical to compound No. 1 (original publication) in Utkina et al., 1987 [41], which was recently reisolated [55]. Compound source of the literature comparison (all the compound numbers were mentioned in the original publication): Utkina et al., 1987 [41]—compound No. 1 (CDCl_3_) and Utkina et al., 2019 [55]—compound No. 3 (DMSO-*d*_6_); Salva and Faulkner 1990 [50]—compound No. 1 (CDCl_3_). Compound **2b** has also been isolated by Handayani et al., 1997 [30], Zhang et al., 2008 [37], and Suyama et al., 2010 [40]. However, chemical shift comparison could not be performed due to the lack of ^1^H and ^13^C NMR data for the related compounds. Therefore, to achieve a close comparison, we used compound 2a from Salva and Faulkner [42], which is similar to compound 2b with an identical A-ring.

**Table 6:** ^13^C NMR data of compound **2b** compared to predicted values and literature data for similar compounds.

Position	δ^13^C [ppm]				
	2b (This Work)	2b *	Utkina et al., 1987 2b	Salva and Faulkner 1990 2a	2a *
1	146.8	147.3	148.8	147.3	147.3
2	139.2	139.8	139.8	139.5	139.5
3	121.0	120.9	117.2	120.9	120.9
4	125.6	120.1	125.2	125.5	120.1
5	119.7	119.4	119.8	119.4	119.4
6	114.2	114.0	115.8	114.0	114.0
1′	151.9	152.2	152.0	152.4	152.4
2′	112.7	112.4	112.1	111.6	111.5
3′	136.2	135.7	135.0	134.0	133.9
4′	115.7	116.1	114.3	124.6	125.3
5′	131.4	131.4	131.3	128.6	128.6
6′	116.0	116.9	116.1	114.5	114.5

Predicted chemical shift values for 2a * and 2b * were added to clarify the structure of our work. An asterisk (*) represents the calculation result of the ^13^C chemical shift values using NMRPredict. Compound **2b** (this work, solvent: CDCl_3_) was identical to Utkina et al., 1987 [41]—compound No. 1 (DMSO-*d*_6_), which was recently reisolated [55]. Compound **2a** [42] was used as a comparison for structure **2b,** which differs by one less bromine at C-4′ in the B-ring. Compound **2b** has also been isolated by Handayani et al., 1997 [30], Zhang et al., 2008 [37] and Suyama et al., 2010 [40]. However, a chemical shift comparison was not included due to a lack of ^1^H and ^13^C NMR data.

## Data Availability

The data presented in this study are available upon request from the corresponding authors.

## Supplementary Materials

The following are available online at https://www.mdpi.com/article/10.3390/md19110611/s1, Figure S1a. The HRMS (a,b) and MS/MS spectra (c) of the compound **1d**. Figure S1b. The HRMS (a,b) and MS/MS spectra (c) of the compound **2b**. Figure S2. Calibration curves of compounds **1d** (a) and **2b** (b) showing correlation between observed peak areas and injected compound concentrations (0.1 µg/mL, 1 µg/mL, 10 µg/mL, 100 µg/mL, 1 mg/mL). Figure S3. HPLC chromatogram from the crude extract of *Lamellodysidea herbacea*. It shows that compounds **1d** and **2b** are the major metabolites of the extract. Figure S4. UV chromatogram of compounds **1d** and **2b** (a,c), including their UV spectra and λmax [nm] (b,d). Table S1. ^13^C NMR data for compound **1d** in CDCl_3_ compared to their chemical shift calculations by two NMR prediction programs NMRShiftDB and CSEARCH.
