# Supplementary material for: Ecological and Pharmacological Activities of Polybrominated Diphenyl Ethers (PBDEs) from the Indonesian Marine Sponge Lamellodysidea herbacea
Source: Mar Drugs. 2021 Oct 27;19(11):611. doi: 10.3390/md19110611 (PMC8621810; doi:10.3390/md19110611)
Supplement: Supplementary file 1 [file marinedrugs-19-00611-s001.zip › marinedrugs-1387733-supplementary.pdf]

SUPPLEMENTARY MATERIAL: Ecological and Pharmacological Activities of Polybrominated Diphenyl Ethers (PBDEs) From the Indonesian Marine Sponge *Lamellodysidea herbacea*

Muhammad R. Faisal, Matthias Y. Kellermann, Sven Rohde, Masteria Y. Putra, Tutik Murniasih, Chandra Risdian, Kathrin I. Mohr, Joachim Wink, Dimas F. Praditya, Eike Steinmann, Matthias Köck and Peter J. Schupp

Figure S1a. The HRMS (a,b) and MS/MS spectra (c) of the compound **1d**.

Figure S1b. The HRMS (a,b) and MS/MS spectra (c) of the compound **2b**.

Figure S2. Calibration curves of compounds **1d** (a) and **2b** (b) showing correlation between observed peak areas and injected compound concentrations (0.1 µg/mL, 1 µg/mL, 10 µg/mL, 100 µg/mL, 1 mg/mL).

Figure S3. HPLC chromatogram of the crude extract of *Lamellodysidea herbacea*. It shows that compounds **1d** and **2b** are the major metabolites of the extract.

Figure S4. UV chromatogram of compounds **1d** and **2b** (a,c), including their UV spectra and λmax [nm] (b,d).

Table S1. ^{13}C NMR data for compound **1d** in $CDCl_3$ compared to their chemical shift calculations by two NMR prediction programs NMRShiftDB and CSEARCH.

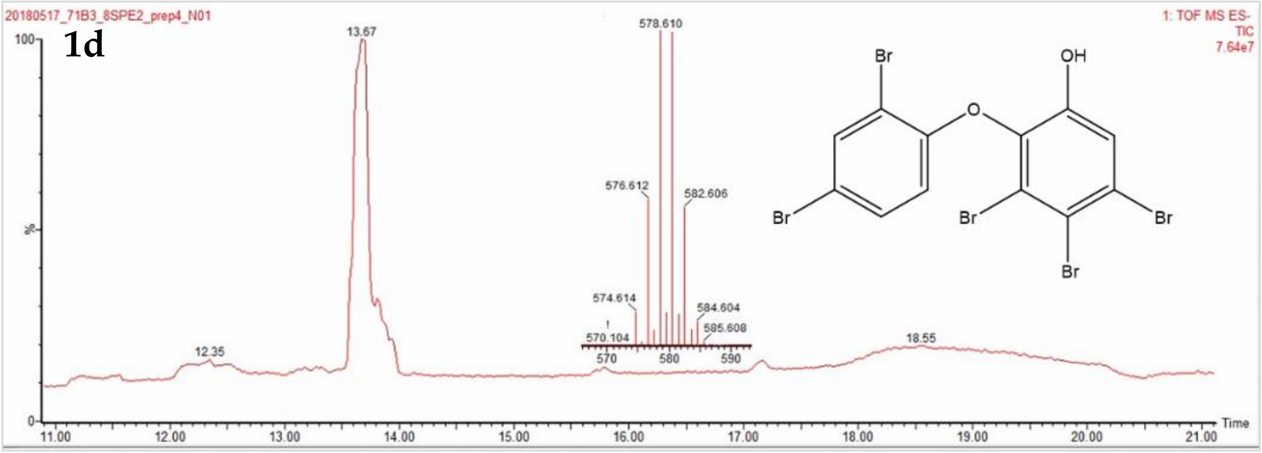

(a)

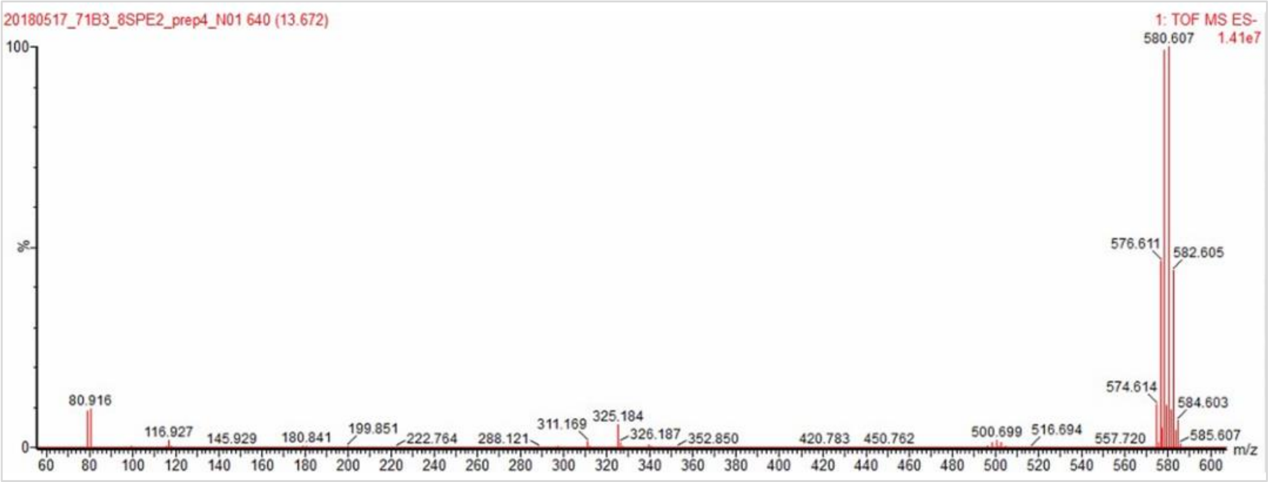

(b)

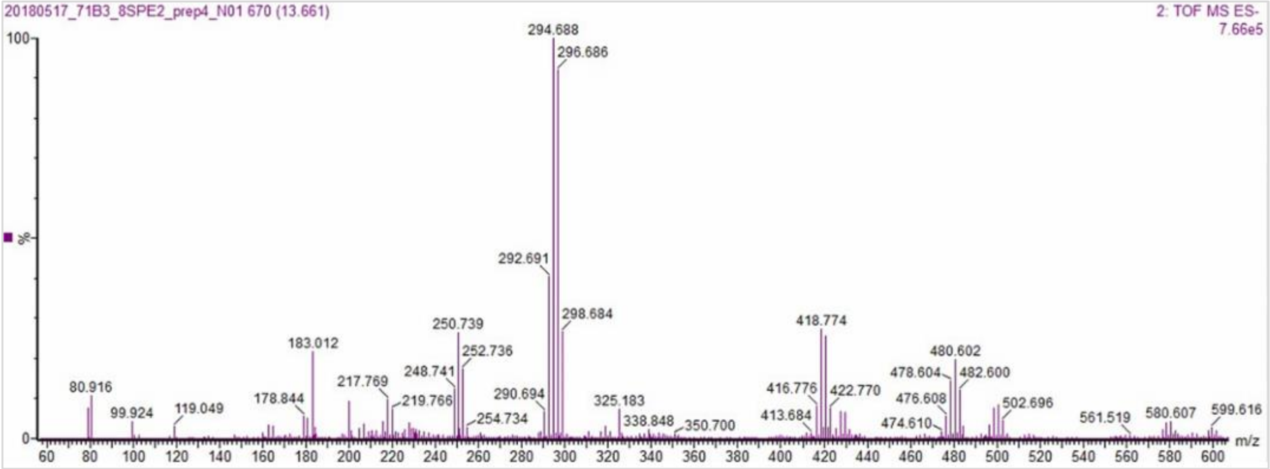

(c)

Figure S1a. The HRMS (a,b) and MS/MS spectra (c) of the compound **1d.**

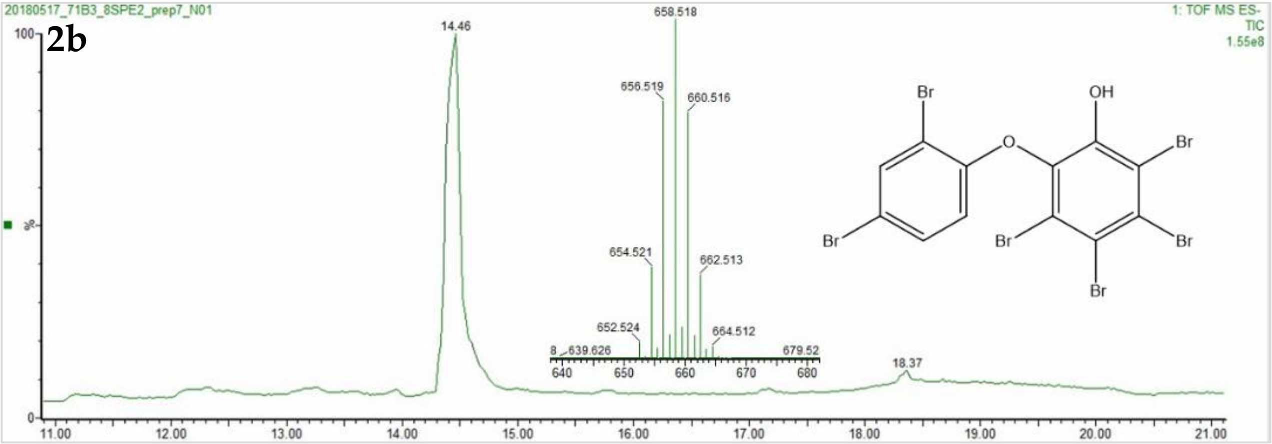

(a)

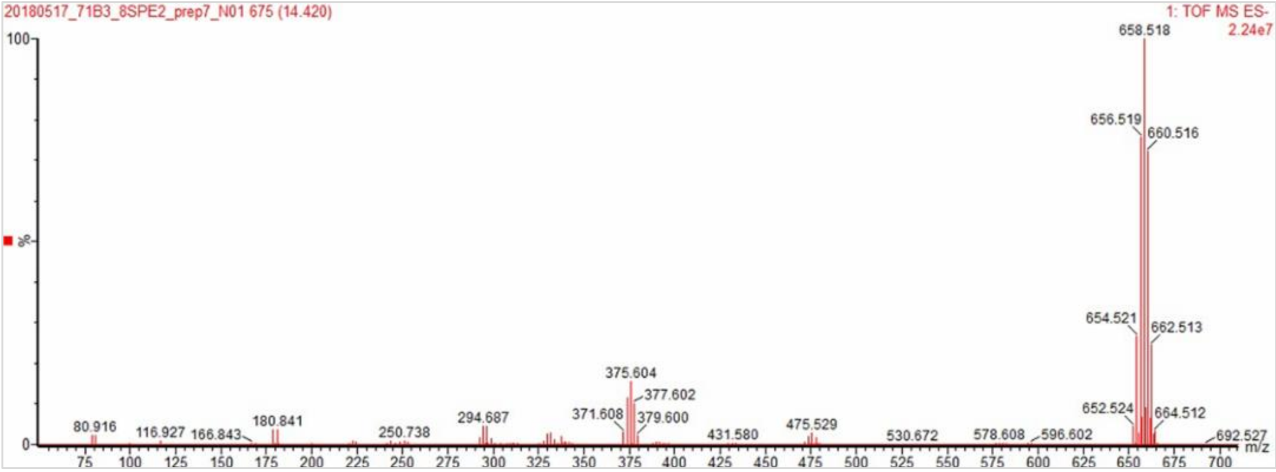

(b)

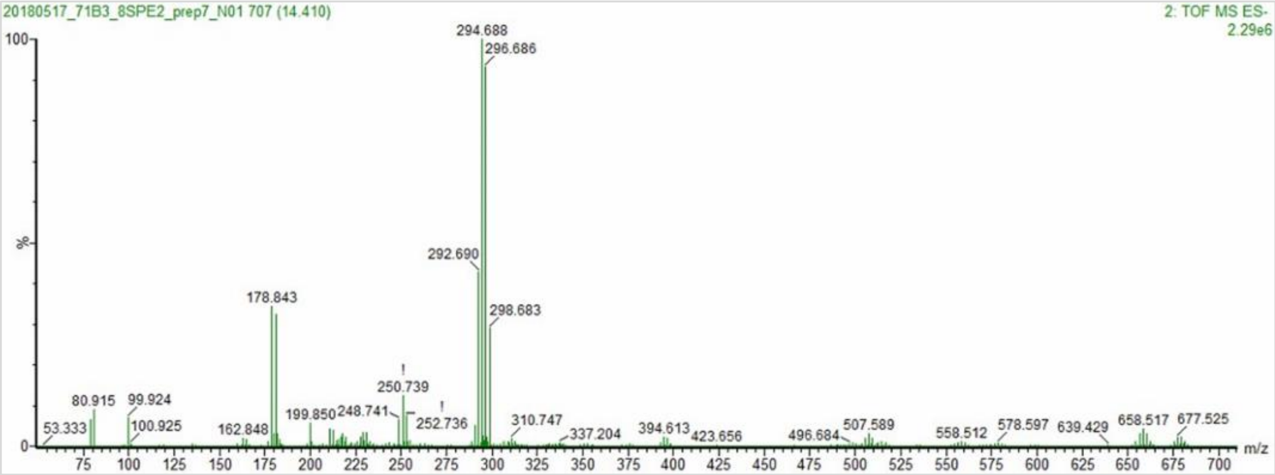

(c)

Figure S1b. The HRMS (a,b) and MS/MS spectra (c) of the compound **2b.**

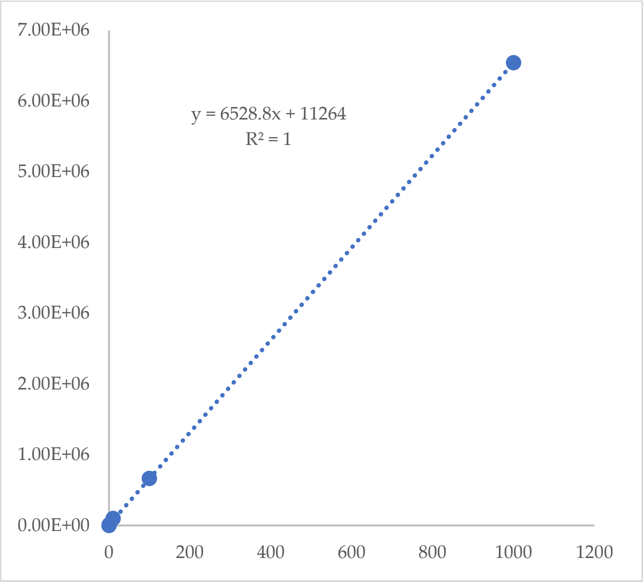

(a)

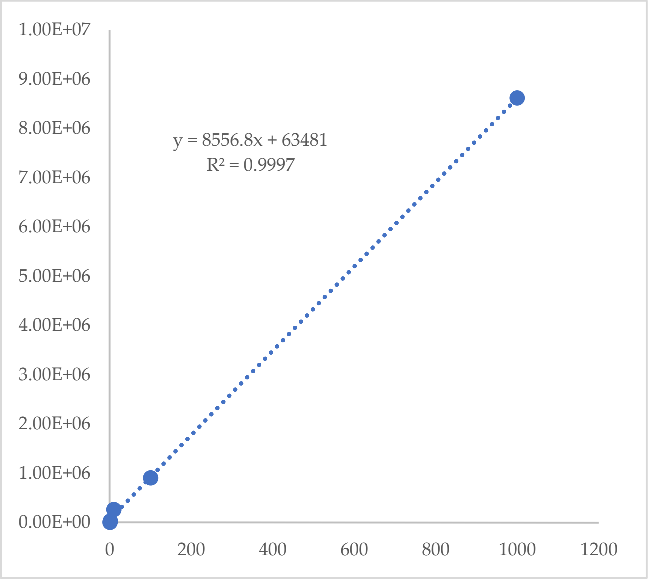

(b)

Figure S2. Calibration curves of compounds **1d** (a) and **2b** (b) showing correlation between observed peak areas and injected compound concentrations (0.1 µg/mL, 1 µg/mL, 10 µg/mL, 100 µg/mL, 1 mg/mL).

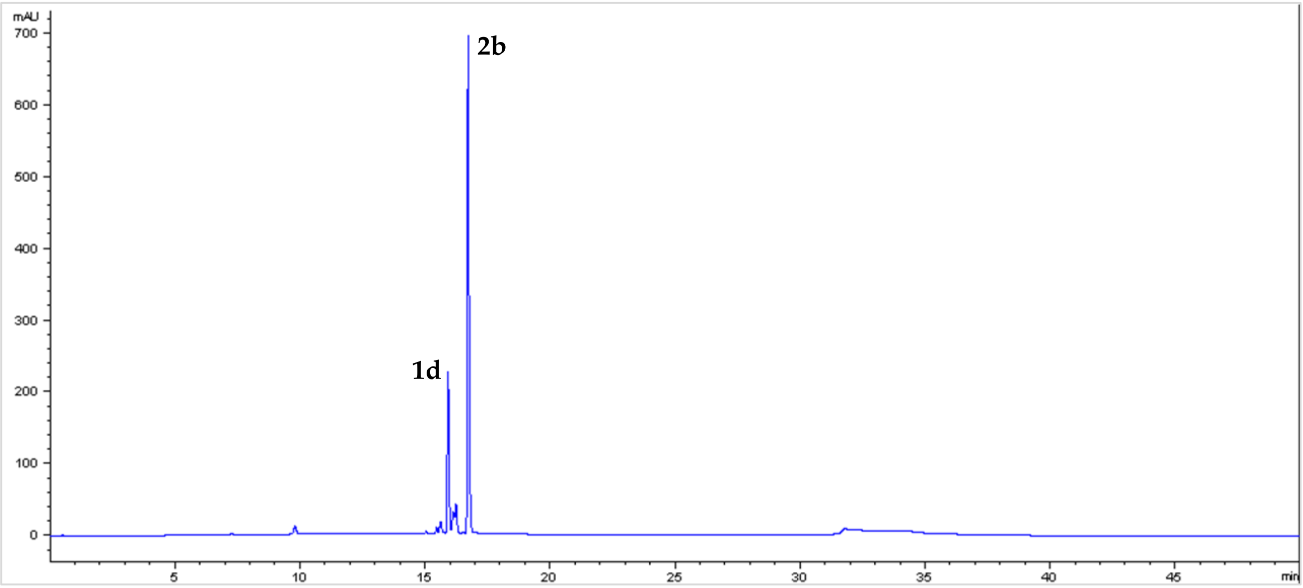

Figure S3. HPLC chromatogram of the crude extract of *Lamellodysidea herbacea*. It shows that compounds **1d** and **2b** are the major metabolites of the extract.

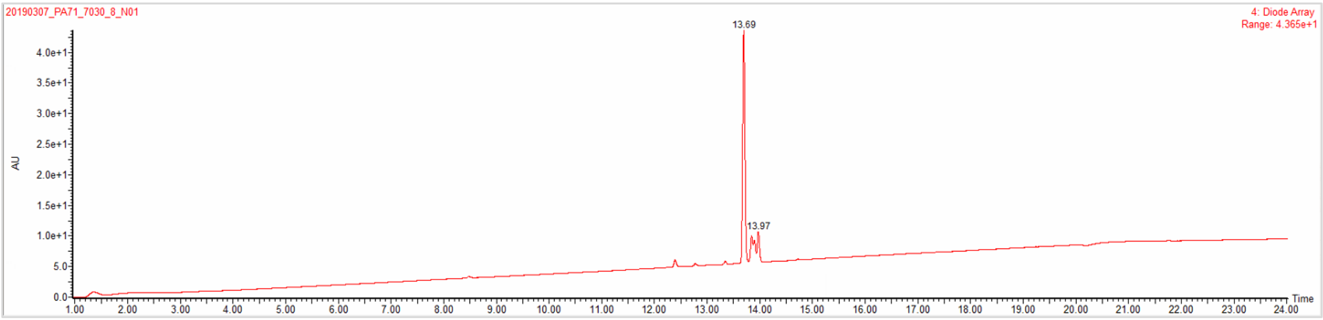

(a)

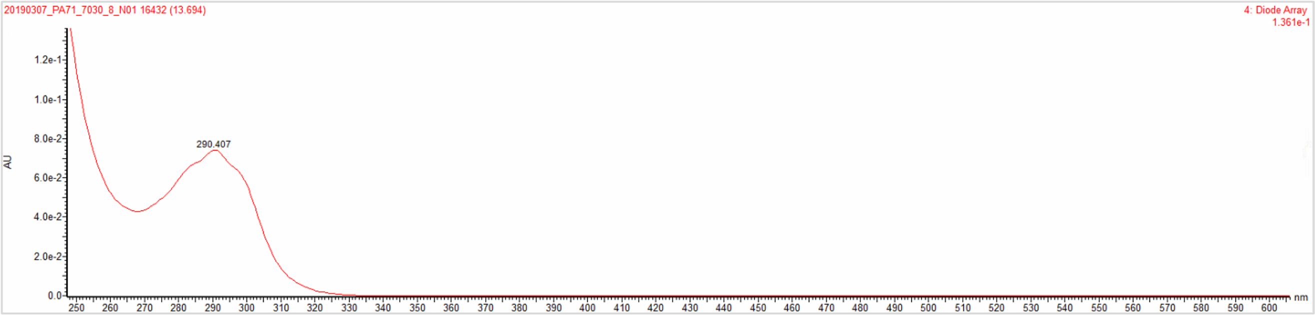

(b)

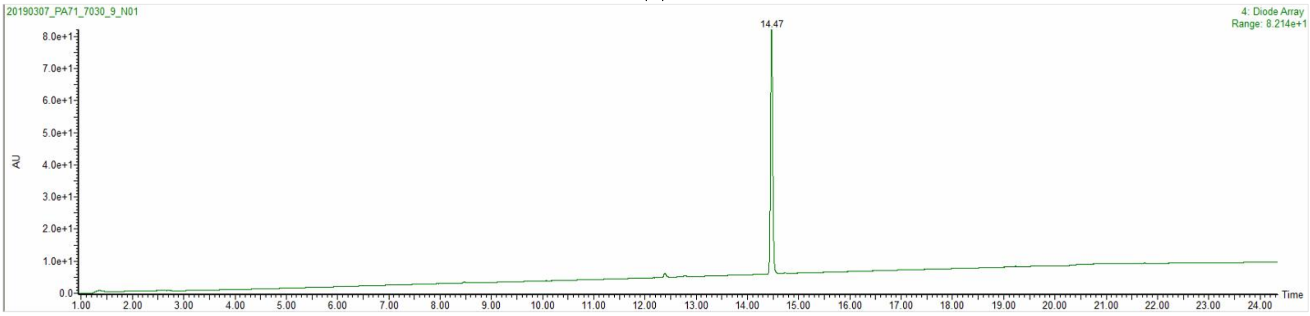

(c)

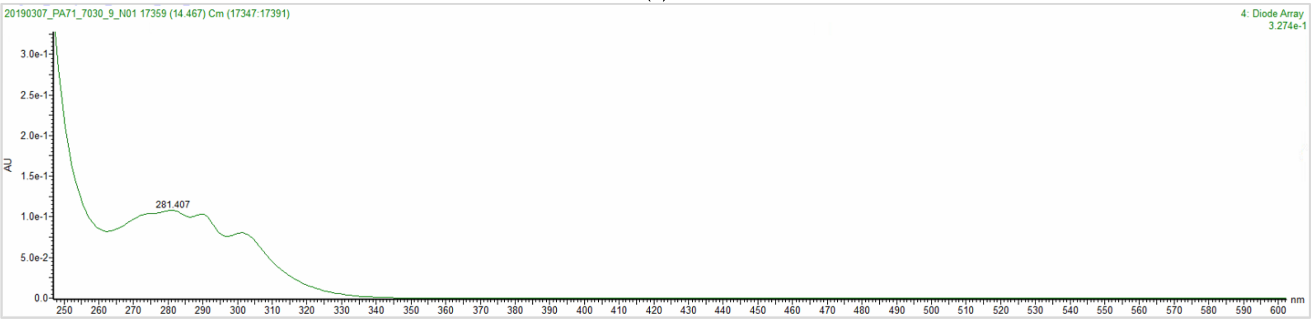

(d)

Figure S4. UV chromatogram of compounds **1d** and **2b** (a,c), including their UV spectra and λmax [nm] (b,d).

Table S1. ^{13}C NMR data for compound **1d** in $CDCl_3$ compared to their chemical shift calculations by two NMR prediction programs NMRShiftDB and CSEARCH.

**Position**	**δ13C [ppm]**								
	**1d** – $CDCl_3$	**NMRShiftDB**				**CSEARCH**			
		**1a**	**1b**	**1c**	**1d**	**1a**	**1b**	**1c**	**1d**
1	148.9	144.37	144.37	144.37	146.74	147.4	148.3	146,6	150.1
2	139.5	148.95	145.52	145.52	145.52	143.5	138.3	140.5	139.7
3	121.1	113.60	110.20	114.70	120.40	121.4	116.8	120.2	119.1
4	119.2	120.40	135.01	124.93	127.88	117.3	127.7	114.7	118.1
5	122.9	127.88	127.88	135.01	123.46	122.6	122.5	133.0	122.5
6	120.7	114.70	114.70	111.12	119.00	122.1	114.0	111.3	120.9
1'	151.7	152.90	152.90	152.90	152.90	151.7	152.3	151.9	152.0
2'	112.7	114.90	114.90	114.90	114.90	115.6	112.8	112.8	112.8
3'	136.3	136.20	136.20	136.20	136.20	136.0	136.1	136.1	136.1
4'	116.2	116.40	116.40	116.40	116.40	116.2	116.2	116.2	116.2
5'	131.6	116.40	131.60	131.60	131.60	132.6	131.7	131.7	131.7
6'	115.7	120.10	120.10	120.10	120.10	121.6	115.9	116.3	116.3

The chemical shift of ^{13}C for compound **1d** (this work) measured in $CDCl_3$ was compared with the publicly accessible NMRShiftDB database (https://nmrshiftdb.nmr.uni-koeln.de/) and CSEARCH (https://nmrpredict.orc.univie.ac.at/c13robot/robot.php).
